# Proteins Associated with Neurodegenerative Diseases: Link to DNA Repair

**DOI:** 10.3390/biomedicines12122808

**Published:** 2024-12-11

**Authors:** Svetlana N. Khodyreva, Nadezhda S. Dyrkheeva, Olga I. Lavrik

**Affiliations:** 1 Institute of Chemical Biology and Fundamental Medicine, Siberian Branch of Russian Academy of Sciences, 8 Akad. Lavrentyeva pr., Novosibirsk 630090, Russia; dyrkheeva.n.s@gmail.com; 2 Faculty of Natural Sciences, Novosibirsk State University, 2 Pirogova Str., Novosibirsk 630090, Russia; 3 Sechenov Institute of Evolutionary Physiology and Biochemistry, Russian Academy of Sciences, 44 Thorez pr., St. Petersburg 194223, Russia

**Keywords:** alpha synuclein, amyloid beta, base excision repair, C9orf72, direct DNA repair, FUS, homologous recombination, huntingtin, liquid–liquid phase separation, mismatch repair, neurodegenerative disease, nonhomologous end joining, NONO, poly(ADP-ribose) polymerase 1, SFPQ, tau protein, TDP-43

## Abstract

The nervous system is susceptible to DNA damage and DNA repair defects, and if DNA damage is not repaired, neuronal cells can die, causing neurodegenerative diseases in humans. The overall picture of what is known about DNA repair mechanisms in the nervous system is still unclear. The current challenge is to use the accumulated knowledge of basic science on DNA repair to improve the treatment of neurodegenerative disorders. In this review, we summarize the current understanding of the function of DNA damage repair, in particular, the base excision repair and double-strand break repair pathways as being the most important in nervous system cells. We summarize recent data on the proteins involved in DNA repair associated with neurodegenerative diseases, with particular emphasis on PARP1 and ND-associated proteins, which are involved in DNA repair and have the ability to undergo liquid–liquid phase separation.

## 1. Introduction

**Figure 1:**
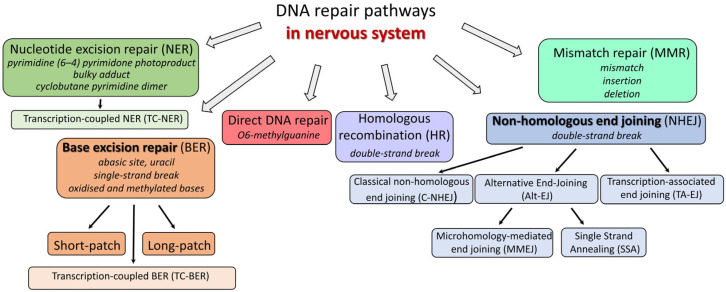
DNA repair pathways and typical repaired lesions (italicized).

The cell, the basic unit of our body, is constantly exposed to various forms of stress. DNA in cells suffers damage from both external and internal sources. There are several types of DNA damage, such as base modifications (bulky and nonbulky), abasic sites, strand breaks (single or double), intra- and interstrand cross-links in DNA, protein-DNA adducts, and mismatches. On any given day, even in the absence of external influences, tens of thousands of deaminated cytosines, hundreds of methylated purines, tens of thousands of spontaneous depurination events, tens of thousands of single-strand breaks, up to hundreds of thousands of oxidative damages, and dozens of double-strand breaks are generated in a mammalian cell [1,2,3,4]. Damage can occur in both the nuclear and mitochondrial genomes. Cells spend enormous resources on genome maintenance and have evolved at least six sophisticated systems to cleanse DNA from lesions (Figure 1). Some DNA repair pathways are only compatible with DNA replication mechanisms and therefore could not operate in neurons, which are terminally differentiated post-mitotic cells.

Reactive oxygen and nitrogen species generated during normal physiological processes, including O_2_ metabolism, immune response, and inflammation [5,6,7], are the major source of endogenous DNA damage. Mitochondria, being the power engine of the cell, are the main site of reactive oxygen species (ROS) generation during the functioning of the mitochondrial electron transport chain [8]. 

Proximity to the source of ROS, coupled with the absence of protective histones, renders mitochondrial DNA the most susceptible to oxidative damage [9]. In fact, the mutation rate of human mitochondrial DNA is about 100 times higher than that of nuclear DNA [10].

To counteract the harmful consequences of DNA damage, cells activate the DNA damage response (DDR). The DDR involves not only the detection and repair of DNA damage but also DNA damage signaling to coordinate DNA repair with other cellular processes [11]. It is worth noting that each type of DNA lesion has its own specific mechanism of recognition and repair. However, in some cases, the same lesions can be repaired by alternative pathways, depending on the state of cell differentiation, cell cycle phase, and tissue.

The nervous system (NS) includes both nondividing and dividing cells. Specifically, neurons are mature, terminally differentiated cells that can no longer undergo mitosis and are therefore defined as post-mitotic. They continue to function throughout the life of the organism. Glial cells (e.g., astrocytes, oligodendrocytes, and microglia) can be in either a proliferative or nonproliferative state.

Brain cells are fairly well protected from external insults by the cranium and the blood–brain barrier (BBB), making endogenous DNA damage the most critical threat to the stability of nervous system cell genomes [12]. The nature of internal sources can be both physiological and pathological [13,14,15]. Our knowledge of DNA damage response and repair mechanisms in terminally differentiated replicative quiescent cells, such as neurons, is very limited. The long-lived, highly metabolically active neurons may require a specialized mode of genomic maintenance.

The specificity of the NS cells’ functioning consists in extremely high physiologic activity, which requires an elevated level of energy production and, therefore, high consumption of oxygen. In addition, the brain tissue is characterized by a high content of easily oxidizable polyunsaturated fatty acids, an increased level of iron, a potent ROS catalyst, and a relative insufficiency of antioxidants, which makes the brain vulnerable to DNA damage [16]. Indeed, the brain tissue, accounting for about 2–3% of total body mass, consumes 20% of the body’s basal oxygen input [17].

Thus, oxidative stress caused by ROS is one of the major sources of DNA damage that represents a threat to the maintenance of long-lived nonproliferative neurons in NS [18]. Mitochondrial dysfunction has been identified as a hallmark of brain degeneration [18,19]. While it is assumed that DNA repair processes in NS are generally similar to those in other cell types, there are many subtle differences, mainly due to the terminal differentiation of neurons, as the ability to repair DNA in nonreplicating NS cells is limited. The aim of this review is to accumulate and expand the knowledge in the field of DNA repair in NS, in particular the base excision repair and double-strand break repair pathways, which are most prominent in NS cells. In this review, we summarize recent data on the process of membraneless organelle (MLO) formation by liquid–liquid phase separation (LLPS), which influences DNA repair and the development of neurological diseases (NDs). We also take a closer look at the role of proteins associated with NDs in DNA repair, with a particular focus on PARP1. It is well known that most NDs are proteinopathies characterized by the accumulation of protein aggregates composed of misfolded proteins. In this review, we also analyze the existing literature on ND-associated proteins that are involved in DNA repair and have the ability to undergo LLPS.

## 2. DNA Repair Pathways

The specificity of the NS cells, namely the post-mitotic state, high metabolic activity, and long life span, determines the distinctive features of DNA repair pathways in neurons as compared with other somatic cells. The cells of NS appear to evolve a unique set of mechanisms to mediate genome maintenance, which are adapted to their functional requirements.

### 2.1. Base Excision Repair (BER)

Base excision repair is the main nuclear and mitochondrial DNA repair pathway aimed at processing non-helix-distorting base lesions resulting from oxidation, alkylation, deamination, and the action of some physical agents [20,21,22,23,24]. Oxidative lesions are the predominant form of DNA damage in the cells of NS, invariably arising in these cells during normal physiological activity. In addition, increased levels of oxidative damage in genomic DNA are associated with the development of some neurodegenerative diseases and aging of the organism [22,23,24]. Overall, this requires efficient functioning of the BER throughout the life of the organism. However, microarray analysis of several regions of the cerebral cortex of humans (20–99 years old) revealed that the expression levels of key BER genes are largely downregulated during aging [25].

**Figure 2:**
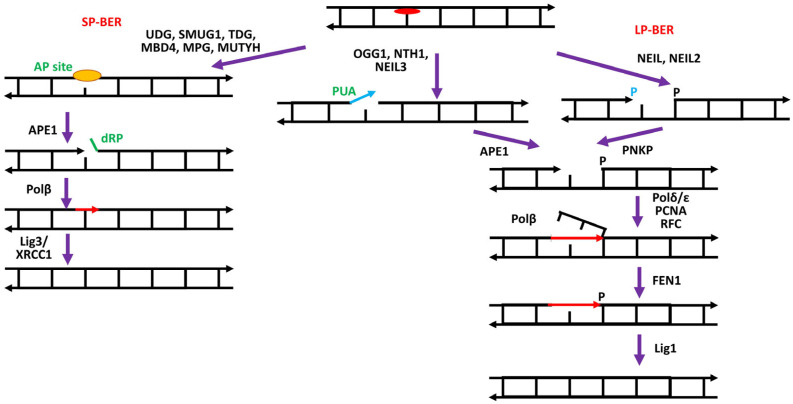
Base excision repair (BER) mechanisms. Base excision repair is performed by short patch (SP-BER) or long patch (LP-BER). Damaged bases are removed by monofunctional (UNG, TDG, SMUG1, MBD4, MPG, MUTYH) and bifunctional (NTH1, OGG1, NEIL1, NEIL2, NEIL3) DNA glycosylases. AP (apurinic/apyrimidinic) sites remaining after the action of monofunctional glycosylases are incised by apurinic/apyrimidinic endonuclease 1 (APE1). dRP (5′ deoxyribose phosphate) is removed by the 5′dRP lyase activity of DNA polymerase β (Polβ), followed by Polβ-catalysed incorporation of a dNMP (SP-BER). The resulting nick is sealed by DNA ligase 3 (Lig3)-XRCC1. Oxidized DNA bases are processed by bifunctional DNA glycosylases, which remove the base and cut into the DNA backbone, creating the nick with 3′ α,β-4-hydroxypentene-2-al (PUA) or phosphate (P). The 3′ PUA residue and the 3′ P group are removed by APE1 and polynucleotide kinase phosphatase (PNKP), respectively. In LP-BER, a 2 to 13 nucleotide patch is synthesized by Polδ/ε (or Polβ) with the assistance of PCNA. A resulting 5′ flap is removed by flap endonuclease 1 (FEN1), and the final ligation step is performed by DNA ligase 1 (Lig1). Red arrow indicates the newly incorporated nucleotide(s); red and yellow ovals indicate the damaged base and AP site, respectively.

The BER pathway (Figure 2) requires four main types of enzymes: DNA glycosylases, apurinic/apyrimidinic endonuclease, DNA polymerase(s), and DNA ligases [20,26,27,28,29,30]. In addition, some accessory/regulatory proteins are also required [30,31,32].

The main strategy of the BER process consists in the conversion of a wide range of substrates with modified bases into a few intermediates that can then be processed by the core BER components. Recognition of the lesions is primarily mediated by enzymes called DNA glycosylases, which are specialized in detecting distinct modified bases and excising them by cleavage of the N-glycosidic bond [33]. The removal of the damaged DNA element as a base is a characteristic feature of the BER process, in contrast to all other repair pathways where the lesion is removed either as a nucleotide or even as a single-stranded DNA fragment.

DNA glycosylases are a heterogeneous group of enzymes that differ in structure, specificity, and mechanism of action. DNA glycosylases are mechanistically divided into two types—monofunctional and bifunctional [22,34,35,36,37].

Monofunctional DNA glycosylases include UNG, TDG, SMUG1, MBD4, MPG, MUTYH and bifunctional ones comprise NTH1, OGG1, NEIL1, NEIL2, NEIL3. The specificity of DNA glycosylases functioning beyond DNA repair in the nervous system is reviewed in [34].

The action of a monofunctional DNA glycosylase results in the formation of the first BER intermediate, DNA containing an apurinic/apyrimidinic (AP) site. In classical BER, the AP site is then hydrolyzed by apurinic/apyrimidinic endonuclease 1 (APE1), resulting in the appearance of the next intermediate—a single-strand break flanked by a hydroxyl group at the 3′ end and a 2′-deoxyribose-5′-phosphate (dRP) residue at the 5′ end [25,38].

Bifunctional DNA glycosylases are specialized in the removal of oxidized bases, the major damage that occurs in the genomes of NS cells [8,24]. Neurons contain a large number of mitochondria, which produce ATP required for their activity. In mitochondria, ROS can occur as byproducts of the oxygen reduction reaction during ATP production.

Bifunctional DNA glycosylases can cleave the sugar–phosphate backbone on the 3′ side of the AP site after excision of the damaged bases, forming strand breaks flanked by 3′-α,β-4-hydroxypentene-2-al (3′-PUA) and 5′-phosphate (β-elimination) or with 3′- and 5′-phosphate groups (β,δ-elimination). Interestingly, the efficiency of cleavage of the sugar–phosphate backbone by different bifunctional DNA glycosylases varies widely; for example, OGG1 and NEIL3 function predominantly as monofunctional glycosylases [39,40]. The structure of the 3′ end of strand breaks generated by bifunctional DNA glycosylases is not suitable for repair synthesis by DNA polymerases and requires removal of the blocking groups. APE1 and polynucleotide kinase 3′-phosphatase (PNKP) act downstream of glycosylases in the BER pathway to remove 3′-dRP and 3′-phosphate, respectively. In addition to these classical 3′-end deblocking enzymes, tyrosyl-DNA phosphodiesterase 1 (TDP1), a member of the phospholipase D superfamily [41], may also be involved in this process. TDP1 can assist in DNA repair of lesions induced by a variety of reagents, including etoposide, methyl methanesulfonate, ionizing radiation, bleomycin, and hydrogen peroxide [42]. TDP1 can handle different lesions, including 3′-phosphoglycolate, 3′-PUA, 5′-phosphotyrosine groups, and intact AP sites [43,44].

At the level of DNA resynthesis, the BER process can be divided into ‘short patch’ (SP) and ‘long patch’ (LP) BER pathways, which are carried out by different enzymes. In SP-BER, DNA polymerase β incorporates a dNMP at the site of the damaged unit. The dRP residue is removed from the DNA by 2′-deoxyribose-5′-phosphate lyase activity (dRP lyase) via the β-elimination mechanism. In mammalian cells, this function is mainly carried out by DNA polymerase β [45,46], which has a separate domain responsible for lyase activity in addition to the nucleotidyltransferase domain [47,48]. In addition, NEIL1 and NEIL2 have been demonstrated to cleave the 5′-residue via β-elimination with an efficiency similar to that of Polβ [49]. The formed nick is then ligated by DNA ligase III in complex with the auxiliary protein XRCC1 [30,50].

If, for some reason (e.g., modification of a sugar residue), Polβ is unable to remove the 5′-dRP residues, the BER process switches to the long-patch pathway, in which, after the incorporation of the first dNMP by Polβ, the repair synthesis continues with the replacement of 2–20 nucleotide units by the “replicative” DNA polymerase δ and/or ε with the involvement of the auxiliary factors PCNA and RFC. The DNA region displaced during DNA synthesis is removed by flap endonuclease (FEN1), and the single-stranded nick is ligated by DNA ligase I [51].

Potentially, repair synthesis with strand displacement can be carried out by concerted action of FEN1 and Polβ via the hit-and-run mechanism [52]. In this case, Polβ incorporates a dNMP, resulting in a nicked DNA molecule with the modified 5′ sugar–phosphate group as a 5′ flap. FEN1 then removes a DNA fragment consisting of a nucleotide and a dangling 5′-sugar phosphate, leaving a single nucleotide gap flanked by a 5′-phosphate. The latter structure is the best substrate for Polβ [53]. This pathway “gap translation” results in the exchange of two nucleotide units. In this case, after the incorporation of a nucleotide, the resulting nick is sealed by DNA ligase I [54]. Otherwise, as shown in a system reconstituted from purified proteins and a BER intermediate with a modified 5′-sugar (pTHF moiety), Polβ is capable of DNA synthesis with a strand displacement of more than two nucleotides, followed by cleavage of the formed flap by FEN1 [55].

The choice between the long-patch and short-patch BER mechanisms is still not fully understood. Overall, the balance between SP- and LP-BER depends on the relative concentration of BER enzymes and scaffolding proteins and the persistence of 5′-blocking lesions at the repair site [56]. If the 5′-dRP group in the 5′-dRP intermediate produced by the AP endonuclease can be efficiently removed by the dRP lyase of Polβ, BER proceeds by the short patch mechanism [57,58].

The predominance of the SP or LP pathway may depend on the cell type. In mature neurons, the SP pathway may be the major BER process due to the reduced expression of some LP BER proteins [59]. At the same time, Polβ-catalyzed PCNA-independent LP repair synthesis may occur in post-mitotic brain cells [60]. Polβ, the major DNA polymerase in noncycling neurons, is ubiquitously expressed in all brain regions [61].

#### 2.1.1. Minor Pathways of BER

A recently discovered subpathway of LP-BER in mammalian cells may also serve to remove oxidative lesions [62]. In this process, a 9-nt gap was formed 5′ to the lesion site by the combined action of RECQ1 (DNA helicase of the RecQ family) and the endonuclease ERCC1-XPF in cooperation with PARP1 and replication protein A (RPA). This new gap formation step may be involved in the repair of various DNA lesions, including oxidative and alkylation damage. This gap is then processed by the replicative DNA polymerases δ (ε), FEN1 and Ligase I, with about 20 nucleotides being replaced.

It has been shown that tyrosyl-DNA phosphodiesterase 1 (Tdp1) catalyzes the AP site cleavage, generating breaks with 3′- and 5′-phosphate termini [63]. Removal of the 3′-phosphate is mediated by PNKP. Tdp1 is known to interact stably with BER proteins: Polβ, XRCC1, PARP1, and DNA ligase III [64]. These findings suggest a role for Tdp1 in the novel APE1-independent pathway of the BER process in mammalian cells.

The BER subpathways initiated by NEIL1 and/or NEIL2 glycosylases and independent of APE1 have been identified [65,66]. NEIL1 and NEIL2 generate DNA strand breaks with 3′ phosphate termini. It has been shown that the 3′ phosphate generated by these glycosylases is efficiently removed by PNKP but not by APE1. Both DNA glycosylases are capable of forming tight complexes with other BER proteins. Complexes containing NEIL2, Polβ, PNK, LigIIIalpha, and XRCC1 (but not APE1) were iso-labeled from human cells competent for the repair of 5-OHU in plasmid DNA [65]. Taken together, these findings support the existence of APE1-independent variants of SP-BER in the repair of oxidized base damage.

Despite the existence of several DNA glycosylases recognizing oxidized bases, cells use noncanonical functions of repair proteins to repair oxidative damage. A DNA glycosylase-independent pathway for repairing some types of oxidized bases has been revealed [67,68]. This subpathway relies on the ability of APE1 to cut the sugar–phosphate backbone on the 3′ side of nucleotides carrying some oxidized bases (5,6-dihydro-2′-deoxyuridine, 5,6-dihydrothymine, 5-hydroxy-2′-deoxyuridine, α-2′-deoxyadenosine) to form the 3′-OH terminus required for subsequent DNA synthesis. This subpathway continues as LP-BER, with the oxidized base being removed by FEN1 as part of the formed flap. It is not clear how realistic this process is in vivo, but it seems reasonable that this unusual pathway may be essential under conditions with high levels of oxidized bases.

It should be noted that several backup BER pathways targeted at the processing of oxidative lesions may be particularly important in neuronal cells, where DNA is vulnerable to such damage due to high metabolic activity.

In addition, proteins of the nucleotide excision repair (NER) machinery may be involved in the initiation of oxidative DNA damage repair (see related section).

#### 2.1.2. Transcription-Coupled Base Excision Repair (TC-BER)

The removal of damage from transcriptionally active sequences, which constitute only a small fraction of the human genome, is of even greater importance in long-lived terminally differentiated nondividing cells of the NS, such as neurons, especially given their high energy requirements that inevitably lead to high levels of oxidative damage.

The preferential removal of lesions from the transcribed strands of active genes, termed transcription-coupled repair (TC-NER, see below in Section 2.2), a subpathway of NER, has been well characterized for bulky DNA photoproducts and adducts [69]. This process is initiated when elongating RNA polymerase II stalls at a bulky DNA lesion induced by UV irradiation [69,70].

At the same time, the preferential repair of oxidized bases in the transcribed strands via the BER pathway has long escaped the attention of researchers because most of these modifications are not capable of distorting the structure of the DNA helix to a degree sufficient to block the activity of RNA polymerase II (RNAP II) [71]. The most common lesions, 8-oxo guanine and thymine glycols, do not stop RNAP II [71].

Using a combination of single-cell gel electrophoresis (comet) and fluorescence in situ hybridization (FISH), the preferential removal of 8-oxoGuanine from the transcribed strand has been demonstrated; in human cells, this process requires RNA polymerase II, hOGG1, XPA, CSB and UVSSA, the proteins belonging to the BER and NER DNA repair pathways [72].

In the search for a DNA glycosylase that can potentially initiate the removal of oxidized bases in TC-BER, NEIL2 has been proposed as a candidate because of NEIL2′s association with RNAP II [71,73,74]. This is consistent with the cell-cycle-independent expression of NEIL2 and the preferential excision of the oxidized base in bubble structures, suggesting a specific involvement of NEIL2 in the repair of transcribed DNA [75].

### 2.2. Nucleotide Excision Repair (NER)

The nucleotide excision repair (NER) pathway repairs a wide variety of DNA lesions, particularly damage that distorts the normal helical structure of duplex DNA (reviewed in [76,77,78,79]). The NER process consists of four main steps: (1) damage detection, which includes initial detection followed by damage verification; (2) double incision on both sides of the lesion and removal of the damage-containing oligonucleotide fragment; (3) gap-filling synthesis to restore an intact DNA duplex; and (4) DNA ligation to seal the remaining nick.

In NER repair, DNA damage can be detected in one of two modes. The global genome NER system (GG-NER) searches for damage anywhere in the genome throughout the cell cycle. The second mode is transcription-coupled NER (TC-NER), which is responsible for the preferential repair of lesions in the transcription chain of actively functioning genes [76,77]. Complete repair of lesions requires the concerted sequential action of more than 30 proteins [76,77,78,80]. GG-NER detects the distortions in the DNA double helix caused by the DNA lesions rather than the lesions themselves [76,78,79,81]. In mammalian GG-NER, DNA damage recognition is achieved by the protein heterodimer XPC-RAD23B. The resulting complex, XPC-RAD23B–damaged DNA, is the point of recruitment for the subsequent proteins, TFIIH, XPA, XPF, and XPG, which in concerted action excise the 30 nt fragment containing the lesion.

TC-NER is triggered by RNA polymerase II (RNAPII) that is stalled at a bulky DNA lesion. For a detailed overview of TC-NER (sequential events, proteins involved, and molecular mechanisms), see the reviews in [82,83,84,85]. In brief, TC-NER proceeds by a sequential and orchestrated assembly of the TC-NER complex. The assembly starts with the recruitment of CSB, followed by the sequential binding of CSA and UVSSA (UV-stimulated scaffold A protein). CSA is recruited as a part of the ubiquitin ligase complex composed of several polypeptides, which appears to be one of the key regulators of the TC-NER process [82,83,84,85]. After recruitment, UVSSA becomes the key protein that recruits the next incoming player, TFIIH, a multi-subunit complex that includes XPB (translocase) and XPD (helicase), among other polypeptides. XPA, XPG, RPA, and ERCC1-XPF are then sequentially recruited to form the active TC-NER complex. Two endonucleases, ERCC1-XPF and XPG, perform a double incision, 5′ and 3′ to the lesion, respectively. The incised DNA fragment containing the lesion is removed in complex with TFIIH and XPG, resulting in a gap of approximately 30 nt.

After this stage, the two pathways, TC-NER and GG-NER, converge on one pathway, reparative DNA resynthesis. One of the DNA polymerases (δ or) fills in the resulting gap, and the nick is ligated by DNA ligase 1 or DNA ligase 3-XRCC1.

TC-NER removes DNA lesions from only a small but critical fraction of the DNA, active genes. For more details on the neurological abnormalities associated with defects in the TC-NER, see the reviews [76,82,86].

With regard to the characteristics of NER function in neurons, the repair of UV-induced DNA lesions in terminally differentiated human neurons hNT was compared with that in their progenitor cells NT2 [87]. This well-characterized system allows the study of repair processes in an identical genetic background. Using the NT2-hNT model system, it was shown that upon differentiation, the GG-NER capacity to remove cyclobutene pyrimidine dimers was significantly reduced in hNT neurons compared with their progenitor cells. Testing the capacity of the TC-NER pathway revealed that NT2 cells repaired both strands of active genes rather efficiently, with the transcribed strand being repaired better than the nontranscribed strand. Such a strand bias is typical and occurs because lesions in transcribed DNA strands, which are substrates in GG-NER, are additionally repaired by the TC-NER machinery. In genes active in hNT neurons, the transcribed strands are repaired, as well as in NT2 cells, suggesting that TC-NER is competent in these cells. Surprisingly, the nontranscribed strand was also found to be well repaired in hNT neurons, with an efficiency comparable to that in NT2 cells. The observed up-regulation of the two structure-specific nucleases, XPG and XPF/ERCC1, correlated with these changes in repair during neuronal differentiation.

Later, for the same cellular model, it was shown that the capacity of NER increases as cell differentiation progresses [88]. RNA-Seq-based transcriptomic analysis revealed that the expression levels of the core NER factors, XPA, XPC, XPG, and XPF-ERCC1, are progressively increased during differentiation into neurons, while those of RPA and TFIIH are down-regulated. The observed discrepancies between these studies [87,88] may be related to different experimental designs, but for two factors, XPG and XPF/ERCC1, the data on up-regulation in differentiated neuron-like hNT cells are consistent. Taken together, these data indicate that at least the TC-NER pathway is preserved in post-mitotic neuron-like cells.

Oxidative DNA damage, the most common lesions generated in neuronal DNA, are generally cleared by BER (reviewed in [89]). However, some ‘bulky’ oxidative lesions, such as 8,5′-cyclo-2′-deoxyadenosine and 8,5′-cyclo-2′-deoxyguanosine, are thought to be repaired by NER (reviewed in [76,90]). Recent studies show that some guanine oxidation products, spiroiminodihydantoin (Sp), 5-guanidinohydantoin (Gh), and certain intrastrand cross-linked lesions, are good substrates for both NER and BER pathways and compete with each other in human cell extracts [90].

The oxidation of guanine by peroxynitrite generates 5-guanidino-4-nitroimidazole (NIm), which is structurally similar to Gh, except that the 4-nitro group in NIm is replaced by a keto group in Gh. However, unlike Gh, NIm is an excellent substrate of BER but not of NER [90].

Accumulating evidence suggests that DNA repair factors may be involved in multiple DNA repair pathways. The BER and NER proteins can assist each other or compete for the same oxidative substrates [91]. For example, the NER proteins XPA, XPC, XPG, CSA, CSB, and UV-DDB act to stimulate glycosylases involved in the removal of certain oxidatively damaged bases in both the nucleus and mitochondria [76,91,92]. At the same time, the BER and NER pathways may compete for the repair of specific products of guanosine oxidation [76,90,91]. Thus, the repair of oxidative DNA lesions by the NER pathway or with the participation of NER proteins may be much more extensive than previously thought.

### 2.3. Double Strand Break Repair (DSBR)

Double-strand break repair (DSBR) is uniquely important for nondividing post-mitotic cells of the nervous system. These long-lived cells must maintain an intact genome throughout life under conditions of high metabolic activity.

Together with DNA double-strand breaks (DSBs) caused by exogenous sources, they can be endogenously induced in the genomes of mammalian cells as a result of increased oxidative stress or specifically as variable diversity joining and class switch recombination events in cells of the immune system [93,94,95]. Interestingly, recent studies suggest that DSBs occur as intermediates during normal physiological processes in neurons, such as memory development [96,97,98].

Mammalian cells use several mechanistically distinct pathways to detect and repair chromosomal DSBs. These pathways include homologous recombination (HR) and nonhomologous end joining (NHEJ), which in turn comprise several subpathways. DSBR pathways are characterized by different kinetics and fidelity; they use different sets of proteins to recognize and repair DSBs; they have unequal cell cycle dependencies and contribute unequally to genome maintenance [98,99,100,101,102,103]. The choice of DSBR pathway mainly depends on the cell cycle phase. It may be that DSBs are particularly dangerous for neurons because, in post-mitotic cells, DSBR can only occur through error-prone and potentially mutagenic mechanisms.

#### 2.3.1. Homologous Recombination (HR)

The HR pathway can only occur in dividing cells and is restricted to the late S and G2 phases of the cell cycle. Among the different DSBR pathways, HR is the most accurate. This error-free functioning is achieved through the use of homologous sequences in sister chromatids that flank the break site during cell replication [104]. Notably, the transcription-dependent recruitment of proteins belonging to recombination repair pathways to oxidative DNA lesions in the neuronal genome has been demonstrated, suggesting that an RNA-templated HR repair mechanism may exist outside of actively replicating cells [105,106].

#### 2.3.2. Nonhomologous End Joining (NHEJ)

**Figure 3:**
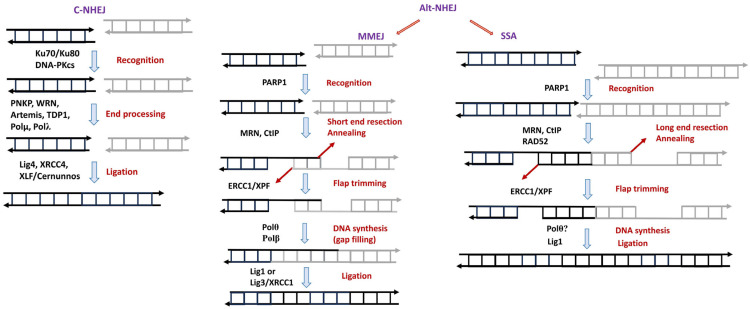
**Nonhomologous end-joining (NHEJ) mechanisms**. NHEJ occurs via classical (C-NHEJ) or alternative (Alt-NHEJ) pathways. Alt-NHEJ is subdivided into microhomology-mediated end-joining (MMEJ) and single-strand annealing (SSA) pathways. In C-NHEJ, DSB recognition is carried out by the Ku70/Ku80 protein, followed by recruitment of the catalytic subunit of DNA-dependent protein kinase (DNA-PKcs), PNKP, and nucleases (WRN or Artemis) or tyrosyl DNA phosphodiesterase 1 (TDP1) and DNA polymerases (Polμ or Polλ) to process the ends as required. DNA ligase 4 (Lig4) rejoins DNA ends in the presence of XRCC4, XLF/Cernunnos proteins. In MMEJ, PARP1 performs recognition and recruits MRN (Mre11/Rad50/Nbs1) and CtIP for short-end resection. After microhomology-mediated annealing of the DNA chains, ERCC1/XPF nuclease trims the gaps. Gaps are filled by Polθ or Polβ, and nicks are sealed by Lig1 or Lig3/XRCC1. In SSA, after long-end resection, RAD52-mediated annealing and ERCC1/XPF-mediated flap trimming followed by DNA synthesis (Polθ), Lig1 seals the nicks. Red arrows indicate unpaired regions of DNA strands.

NHEJ (Figure 3) is the only DSB repair pathway that normally operates in all phases of the cell cycle. The function of classical nonhomologous end joining (C-NHEJ) relies on the set of core NHEJ proteins, including Ku70/Ku80, DNA-dependent protein kinase catalytic subunit (DNA-PKcs), DNA ligase IV, XRCC4, XLF/Cernunnos, and Artemis; C-NHEJ is considered to be the major DSBR pathway in mammalian cells [98,100,101]. The efficiency of C-NHEJ is maintained at a constant level throughout the cell cycle and is essential for the elimination of DSBs in the majority of cells, especially in quiescent or terminally differentiated cells such as neurons [98,100,101,107,108].

C-NHEJ starts with the binding of the Ku70/80 heterodimer to DSB ends, followed by the recruitment of DNA-PKcs to form an active DNA-PK holoenzyme that serves as a landing platform for the recruitment of other C-NHEJ factors [109,110]. The generation of DNA ends compatible with ligation may require DNA end processing. The chemistry of the DNA end determines which factors and enzymatic activities are required. Enzymes that contribute to DNA end processing include Artemis nuclease, DNA polymerases µ and λ, TDP1, and PNKP [111,112]

The alternative NHEJ (Alt-NHEJ) pathway, also known as backup nonhomologous end-joining NHEJ, which is typically defined as any form of end joining that does not require the core C-NHEJ proteins [101,113], includes microhomology-mediated end joining (MMEJ) and single-strand annealing (SSA) [98,100,101]. MMEJ and SSA typically utilise SSB repair enzymes/proteins to repair DSBs [98,100,101]. It is generally accepted that in the early stages of MMEJ, poly-ADP ribose polymerase 1 (PARP1) recognizes DSBs, and binding of PARP1 appears to promote limited resection of DNA ends by the Mre11/Rad50/Nbs1 (MRN) and CtIP complex [114,115]. Further annealing of 2–20 nucleotide microhomologies in the 3′-tails occurs with subsequent DNA resynthesis catalyzed by DNA polymerase θ (Polθ) [116] and removal of the unpaired nonhomologous 3′-tails by the nuclease ERCC1/XPF. Gaps within the DNA strands can be filled by Polθ-catalyzed DNA synthesis [116]. DSB ends are rejoined by DNA ligase III/XRCC1 or DNA ligase I [117,118]. Alt-NHEJ is slower than C-NHEJ in all phases of the cell cycle [119]. Alt-NHEJ is considered to be error-prone. During the processing of DSBs by Alt-NHEJ, the probability of unrelated DNA ends being joined is increased.

SSA is a homology-dependent pathway requiring rather extensive resection of DNA ends and RPA displacement to search for complementary homologous sequences. SSA requires RAD52 activity for the annealing step [120,121] and the structure-specific endonuclease ERCC1-XPF to remove unpaired noncomplementary tails [121,122]. The resulting nick is sealed by DNA Ligase I [121,122]. Due to the necessity of a sufficiently long DNA resection, the SSA is able to operate in the S and G2 phases of the cell cycle [120,121,122,123].

Recent evidence suggests that neurons have additional specialized DSB repair mechanisms that operate in the regions of transcriptionally dependent DSB formation. The transcribing RNA polymerase may recruit DNA repair factors to organize the TA-EJ complex to repair such lesions. Transcriptionally associated DSBs arise from two adjacent single-strand breaks on opposite DNA strands. One SSB results from the action of the topoisomerase I cleavage complex (TOP1cc), and the second from the cleavage of R-loop structures, which are RNA/DNA hybrids with displaced single-stranded DNA [71,124,125,126,127]. Further end joining is dependent on BRCA1, Polθ, and DNA ligases 1 and 3, but not DNA ligase 4 or PARP activity, suggesting repair of DSBs at transcriptionally active regions by an end-joining process distinct from canonical non-homologous end joining or known alternative end-joining pathways [127].

In another study, proteins of classical NHEJ were found to form a multiprotein complex with RNAP II and preferentially associate with transcribed genes after DSB induction; depletion of C-NHEJ protein factors strongly abolished the repair of transcription-associated DSBs, but not in nontranscribed genes [124]. The authors hypothesize that nascent RNA can serve as a template to resynthesize the lacking sequences, which allows error-free DSBR [124]. TA-EJ is a multistep process that requires reverse transcriptase (RT) activity to synthesize a DNA strand using RNA as a template. It was later reported that DNA polymerase η, which has RT activity, is involved in TA-EJ [128]. Pol η was shown to form a multiprotein complex with RNAP II and other protein factors while also associating with nascent RNA. Furthermore, Pol η deficiency results in the accumulation of R-loops and the persistence of breaks in transcribed genes.

Alternatively, recruitment of RAD52, the protein involved in homologous recombination, to sites of DSB lesions was found to be dependent on the presence of a nascent mRNA template, indicating the existence of an RNA-templated recombinational repair pathway in post-mitotic neurons [106].

It has recently been reported that huntingtin (Htt) promotes the organization of the TA-EJ complex composed of PNKP, Ku70/80, and XRCC4 with the chromatin remodeler Brahma-related gene 1 (BRG1) and stimulates DSB repair of transcription-associated DSBs in the brain [129]. Specifically, recruitment of Htt to DSBs in transcriptionally active regions was shown to be BRG1-dependent, whereas efficient recruitment of TA-EJ proteins is Htt-dependent. Mutant Htt disrupts interactions within the TA-EJ complex and reduces DSB repair activity, resulting in the accumulation of DSBs in tissues from Huntington’s disease (HD) patients.

### 2.4. Direct DNA Repair

O6-methylguanine (O6-meG) is a major cytotoxic and mutagenic DNA lesion produced by methylating agents [130,131,132]. In cells, this DNA lesion can be circumvented by translesion DNA synthesis, which results in mutations due to the ability of O6-meG to pair with thymine [133].

Most organisms contain a specific enzyme, O6-methylguanine-DNA methyltransferase (MGMT). MGMT can directly transfer the alkyl group from the O6 position of guanine to a cysteine residue in the active site of the enzyme, thereby restoring the undamaged state of guanosine and inactivating the enzyme [134,135]. Inactivated MGMT is degraded by the proteasome following ubiquitination [136].

Even in the absence of exogenous sources of alkylating agents, one would expect an important role for MGMT in long-lived post-mitotic cells of NS, given that spontaneous DNA alkylation products can be generated via reactions with the endogenous S-adenosylmethionine. Even in the absence of exogenous sources of alkylating agents, one would expect an important role for MGMT in long-lived post-mitotic cells of NS, considering that spontaneous DNA alkylation products can be produced via reactions with endogenous S-adenosylmethionine. Given the post-mitotic state of most cells in NS, it is reasonable to expect a more significant effect on cell function through interference with the transcriptional process. At the level of transcription, O6-meG can block human RNA PII or, alternatively, the lesion can be circumvented by incorporating cytosine or uracil opposite it. The latter can result in proteins with altered primary amino acid sequences and, in some cases, defective protein function [134,135].

### 2.5. Mismatch Repair (MMR)

The mismatch repair system recognizes and corrects base–base mismatches and insertion-deletion loops that can occur during DNA replication in dividing cells, but this repair mechanism is not required in neurons because of their nondividing state. Today, MMR deficiency is not clearly associated with any neurological dysfunction in humans; however, it has been reported that MMR may contribute to the expansion of trinucleotide repeats, such as those characteristic of HD, and thus, MMR deficiency in neurons may be associated with neurodegeneration [137]. In addition, TDP-43, a protein associated with neurodegeneration, has been found to disrupt the expression of MMR proteins and to interact with the MMR proteins MLH1 and MSH6 in a DNA-damage-inducible manner [138,139]. Many questions concerning MMR function in neurons still remain to be addressed.

In this part of the review, we have therefore provided an overview of the different DNA repair pathways, focusing on BER and NHEJ, repair pathways that have been shown experimentally to function efficiently in post-mitotic neurons. For each of the repair pathways, we have noted the characteristics of their function in NS cells. Here, we also summarize, in Table 1, data on mutations in DNA repair genes in rodent models of neurodegenerative disease. This information for other animal models can be found, for example, in [140,141,142]. It is interesting to note that while mice with mutations in repair genes may show features of neurological disorders (Table 1), they often do not show neurological phenotypes. This may be due to the shorter lifespan of mice compared with humans. Another consideration is that mutations in repair genes often result in embryonic lethality. Therefore, animal models may not always be good models for human NDs.

In the next chapter of this review, we discuss the phenomenon of liquid–liquid phase separation (LLPS), which has been extensively studied over the last few decades in relation to biological systems, including, as we discuss below, DNA repair.

## 3. Liquid–Liquid Phase Separation (LLPS) as Factor Influencing DNA Repair

Neurodegenerative diseases (NDs) are characterized by progressive loss of function and death of neuronal cells. Despite the diversity of clinical symptoms, NDs share some common pathological features, including the misfolding and aggregation of certain proteins in specific regions of the nervous system. The molecular mechanisms underlying the initial misfolding of proteins and their transition from a functional state to pathological aggregates have not been fully elucidated, despite intensive study in recent years.

Biomolecular condensates, also known as membraneless organelles (MLOs), represent micron-scale compartments in eukaryotic cells that lack surrounding lipid bilayer membranes. Recent work has highlighted the prevalence of cell compartments that are not surrounded by phospholipid membranes. Many different MLOs are found in the nucleus and cytoplasm of eukaryotic cells. Nuclear MLOs include the nucleolus, Cajal bodies, and paraspeckles, whereas cytoplasmic MLOs include stress granules, RNA transport granules, and P bodies [161,162,163]. While many MLOs are found in different cell types, some neuron-specific MLOs have been discovered (reviewed in [162]). The unique membraneless compartments in neurons are represented by the post-synaptic density and elements of the presynapse [162]. These neuron-specific MLOs are characterized by their functional diversity and unique constituent proteins and interactions.

A key mechanism underlying the formation of MLOs is liquid-liquid phase separation (LLPS). LLPS is a phenomenon in which mixtures of two or more components self-segregate into separate liquid phases. It is a special case of a phase transition occurring in a homogeneous solution, a system comprising a solvent (water in the case of biological systems) and homogeneously distributed dissolved substances (e.g., proteins and nucleic acids). This homogeneous solution may undergo, under certain conditions, a spontaneous separation into two immiscible phases, which, respectively, contain more or less of certain dissolved substances [164]. In recent years, the study of LLPS of biomacromolecules has become a frontier in biological research [162,165,166,167]. The formation of biomolecular condensates via LLPS is considered an underlying mechanism of the spatiotemporal coordination of biological activities in cells [168]. Accumulated evidence has shown that LLPS play key roles in regulating various locally performed biological processes, with DNA damage response and DNA repair being among them [169,170]. Emerging evidence suggests that LLPS has a major impact on the regulation of human health and diseases, particularly NDs. LLPS is involved in the regulation of the normal functions of the body and can also lead to abnormal protein aggregation, cytotoxicity, and deterioration of the organism’s functioning.

Biological macromolecules can undergo LLPS under certain conditions, many of which are not common in living cells [162,166,167,171]. The initiation of LLPS is highly dependent on the concentration and physicochemical properties of the biomolecules and the environmental conditions, such as temperature, pH, salt type and concentration, and the surrounding biological macromolecules. In fact, only a small percentage of macromolecules are capable of phase separation in physiological contexts. In recent years, the common characteristics of biomolecules that can undergo LLPS in living cells have become known. Biomolecules capable of phase separation under physiological conditions are usually multivalent and characterized by intra- or intermolecular interactions [167,171]. Proteins that exhibit an enhanced ability to partition into biomolecular condensates can be completely unstructured or hybrids of structured domains and long stretches of intrinsically disordered regions (IDRs). IDRs contain predominantly polar or charged amino acids and lack sufficient hydrophobic residues to mediate cooperative folding. Under physiological conditions, IDRs exist in a dynamic equilibrium of multiple conformational states with varying degrees of folding [172,173]. A significant proportion of proteins prone to phase separation are multifunctional RNA binding proteins (RBPs) [174,175,176]. Liquid–liquid phase separation of RNA-binding proteins is essential for many normal functional processes in cells, but aberrant phase transition of some proteins leads to the formation of insoluble protein aggregates, which are pathological hallmarks of some NDs) [175,176,177,178,179,180].

A propensity for LLPS is characteristic of a number of disordered proteins associated with neurodegeneration [175,176,180,181], with Tubulin Associated Unit proteins (Tau) [182], α-synuclein (αSyn) [183] and amyloid beta (Aβ) [184,185] being the best known.

Another class of biomolecules involved in the formation of MLOs is nucleic acids (RNA and DNA). RNAs, including pre-mRNAs, mRNAs, lncRNAs, miRNAs, and small double-stranded RNAs, are associated with biomolecular condensates [186]. The propensity of nucleic acids to phase separate and the final structure of the formed condensates is determined by several key properties, including their charge, length, sequence, structure, and rigidity [186,187]. Nucleic acids have a highly negatively charged sugar–phosphate backbone, with charged groups evenly distributed along the backbone, allowing them to participate in multiple electrostatic interactions regardless of their sequence. This structural organization promotes their phase separation [186]. The structure of nucleic acids, namely whether they are single-stranded or double-stranded, as well as their length, influence the organization of condensates [186,187]. It should be noted that unlike DS nucleic acids, exposed nucleobases in single-stranded nucleic acids can participate in pi–pi or cation–pi interactions [186,187].

It should also be noted that polyADP-ribose (PAR), the polymer composed of ADP residues formed in response to DNA damage, may also be involved in LLPS [162,181,187,188,189,190,191]. In response to DNA damage, PARP1 promptly detects and binds SSBs and DSBs, leading to PARP1 activation and the synthesis of PAR, which recruits PAR-binding proteins to organize supramolecular DNA repair complexes [188,190,191,192,193]. The same characteristics that define the propensity of nucleic acids for LLPS are also applicable to PAR molecules, including their large negative charge, low complexity, flexibility, and chain length. However, PAR has a unique feature, branching [187]. In addition, PAR has another charge density as compared with single-stranded nucleic acids. The double negative charge of PAR appears to enable it to form more electrostatic contacts and more efficiently interact with protein partners [188].

The assembly of biomolecular complexes at DNA break sites is reversible due to the activity of PAR glycohydrolase (PARG). PARG is the major enzyme involved in the removal of PAR from target proteins, acting both as exo- and endoglycosidase [193,194].

Therefore, in this chapter, we reviewed the phenomenon of LLPS in DNA repair. A growing body of experimental data indicates that reparative processes in cells take place in biomolecular condensates formed by LLPS. At the same time, this phenomenon is also involved in the misfolding and aggregation of a number of ND-associated proteins. In general, there is a need to study these processes in detail. In addition, we are reviewing the data on PARP1 and ND-associated proteins that have the ability to undergo LLPS and have an impact on DNA repair.

## 4. Proteins Associated with Neurodegenerative Disorders

### 4.1. Poly(ADP-Ribose) Polymerase 1 (PARP1)

Among the proteins associated with NDs, PARP1 occupies a special place. PARP1 is widely known for its role in DNA damage repair and other cellular processes such as chromatin remodeling, transcription, and cell death signaling [195,196,197,198]. PARP1 is a pivotal enzyme that connects different pathways of DDR and DNA repair [195,196,197,198,199]. PARP overactivation is a hallmark of some neurological disorders, such as PARP overactivation [200]. In particular, PARP1 plays a dualistic role in the cells of NS, acting on the one hand as a neuroprotector facilitating DNA repair and, on the other hand, inducing cell demise in various neurological disorders [196,200,201,202].

For example, mild DNA damage causes moderate PARP1 activation. This activation leads to the recruitment of proteins involved in DNA repair, including XRCC1 at SSBs and MRE11 and ATM at DSBs, thereby promoting DNA repair [195,196]. In addition, PARP1 promotes DNA repair by relaxing chromatin through the PARylation of histones and organization of biomolecular condensates [165,203,204]. Severe DNA damage leads to PARP1 overactivation, resulting in the depletion of NAD+ and ATP. This overactivation results in the formation of large amounts of PAR polymers which, after being cleaved from the protein by PARG, translocate from the nucleus to the cytosol, inducing the translocation of AIF from the mitochondrial inner membrane. This, in turn, leads to neuronal death (parthanatos) with the involvement of MIF (macrophage migration inhibitory factor) [195,196,205].

Direct interactions have been found between PAR and several proteins: Aβ, TDP-43, and αSyn have been shown to influence the kinetics of protein aggregation and the potential toxicity of these aggregates [201,206,207,208]. For example, in PD, preformed fibrils of αSyn cause PARP1-dependent neurotoxicity by inducing oxidative damage and DNA lesions, which triggers PARP1 overactivation and PAR accumulation, accelerating the development of PD [209].

Recently, the interaction of FUS with PAR has been studied using a variety of approaches [210]. FUS was shown to specifically bind PAR synthesized by PARP1 via its RNA recognition motif (RRM). In cells, FUS increases nuclear PAR levels upon genotoxic stress induced by hydrogen peroxide due to the specific recognition of PAR by the FUS RRM. The increase in PAR levels is promoted by transcriptional arrest due to the release of FUS from nascent mRNAs. Released FUS is directed to activated PARP1 at DNA damage sites through its interaction with PAR. The FUS-dependent increase in PAR levels should promote the formation of fluid-like biomolecular condensates and the recruitment of additional acceptor proteins for PARylation, including FUS itself. Interestingly, the condensates formed by PARylated PARP1 and FUS at DNA damage sites are enriched in damaged DNA, which may facilitate the DNA repair process [193].

Consistent with this, in an in vitro system using purified human PARP1, it was shown that biogenic cations such as Mg^2+^, Ca^2+^, Mn^2+^, spermidine^3+^ or spermine^4+^ induce the assembly of PARylated PARP1 into multimolecular associates on damaged DNA [192]. This association of PARylated PARP1 with repair proteins strongly stimulates Polβ-catalysed strand displacement DNA synthesis but has no appreciable effect on DNA ligase III activity. Using fluorescence-based and light-scattering techniques in an in vitro system containing BER proteins and PARP1, it was shown that PARP1 activation on damaged DNA initiates the formation of the Polβ-XRCC1 complex on PARylated PARP1 [211].

One of the poorly understood aspects of DSB repair is how broken DNA is prevented from separating. Recently, in an in vitro system consisting of purified human PARP1 and DS DNA of different lengths, the formation of DNA co-condensates with PARP1 multimers was demonstrated [212]. Interestingly, PARP2 did not form condensates with DS DNA. The authors proposed a comprehensive model for the hierarchical assembly of DSB condensates to explain DNA end synapsis and the recruitment of effector proteins for DNA repair. The binding of PARP1 to free DNA ends induces protein–protein interactions that promote the assembly of condensates. Within the condensate, DNA-bound PARP1 adopts its catalytically active conformation, spatially confining PAR synthesis to the DNA lesion. PARylation reorganises PARP1-DNA interactions to promote the release of free DNA ends from PARP1. PARylation also promotes the re-recruitment of effector proteins such as FUS, which stabilises broken DNA ends to prevent disjunction of broken DNA ends.

Taken together, the recent studies clearly demonstrate the formation of PARP1 condensates upon DNA binding to single- and double-strand breaks, suggesting the involvement of biomolecular condensates in the organization and regulation of single- and double-strand break repair with the participation of FUS. Further studies are required to elucidate how other proteins associated with NDs may interfere with biomolecular condensates involved in DDR and DNA repair.

### 4.2. Role of Proteins Associated with Neurodegenerative Disorders in the DNA Repair Pathways in Neurons

Most NDs are commonly classified as proteinopathies, characterized by the accumulation of protein aggregates composed of misfolded proteins. These misfolded proteins are known to interfere with various cellular systems by increasing oxidative stress, causing DNA damage, promoting mitochondrial dysfunction and impairing DNA repair [174,195]. As a result, the accumulation of damaged DNA is significantly increased in the cells of patients suffering from NDs [195,213]. Amyloid peptide (Aβ) and tau protein are most commonly associated with Alzheimer’s disease and tauopathies, α-synuclein with synucleopathies, such as Parkinson’s disease or Lewy body dementia, tar DNA-binding protein 43 (TDP-43), FUS and dipeptide repeats with amyotrophic lateral sclerosis (ALS) and/or frontotemporal lobar degeneration (FTLD), and huntingtin (Htt) with Huntington’s disease. Table 2 summarizes ND-associated proteins that have the ability to undergo LLPS and whose involvement in DNA repair has been experimentally confirmed.

#### 4.2.1. FUS

FUS belongs to the FET family of RNA binding proteins (RBPs), which are primarily involved in RNA metabolism [214,218,270]. FUS is involved in the regulation of transcription, pre-mRNA splicing, mRNA transport, and translation. In addition, FUS is involved in the maintenance of DNA integrity [178,179,218,270]. In healthy neurons, FUS is predominantly localized in the nucleus but can shuttle between the nucleus and cytosol in response to various stimuli [214]. FUS is capable of forming diverse structures in vivo, including aggregates, hydrogels, amyloid fibrils, and liquid droplets [214,218]. FUS protein is sequestered in the cytosol of ALS-affected motor neurons.

Although in vitro studies have shown that FUS is able to bind directly to single- or double-stranded DNA [271], how FUS is attracted to sites of DNA damage in cells has not been fully understood. The recruitment of FUS to sites of DNA damage analyzed by the laser microirradiation technique, which is capable of generating the defined DNA damage in a restricted area of the cell nucleus, has revealed the rapid translocation of FUS to DNA-damaged foci [178,216,272,273,274]. FUS recruitment to sites of laser-induced DNA DSBs has been shown to be dependent on PARP activity [216]. In addition, PAR-dependent FUS accumulation at another type of DNA damage, oxidative DNA damage induced by a UVA (320–400 nm) laser, has also been demonstrated [274]. Thus, FUS recruitment may occur not only at DSBs but also at SSBs, which occur at the positions of oxidized bases in the DNA. How FUS and other RBPs are recruited to broken ends remains to be determined. This may occur by binding to PAR, which is synthesized by PARP1 at sites of DNA damage. PARP1 is known to be one of the first proteins recruited to sites of DNA damage [178,275].

FUS is able to directly interact with PAR chains through its RGG domain; the ability to bind PAR can potentially promote LLPS of FUS [191,193], indicating the important role of FUS LLPS in DNA damage response and repair. Indeed, when the interactome of phase-separated FUS was compared with that of non-phase-separated FUS, it was shown that proteins involved in the DNA damage response were almost exclusively detected together with phase-separated FUS [217]. Furthermore, the LLPS of FUS is important for the initiation of DNA damage repair, as LLPS-deficient FUS variants affect the accumulation of DNA repair factors at sites of laser-induced DNA damage.

The depletion of FUS affected both HR and NHEJ, implicating FUS as an upstream participant in both DSB repair pathways [178,216,273]. In particular, FUS has been shown to be required for the recruitment of NBS1 (a component of the DSB-sensing MRN complex), Ku80 (subunit of the Ku antigen), and 53BP1, the proteins involved in the early stages of DSB repair [178]. The translocation of Ku80 and NBS1 was shown to be impaired by LLPS inhibitors or in cells with LLPS-deficient FUS variants [178].

PAR-dependent FUS localization to SSB sites facilitates recruitment of nuclear DNA ligase III/XRCC1 to activate it and thereby increase the efficiency of BER/SSBR [214]. In motor neurons, loss of nuclear FUS caused defects in DNA nick ligation due to reduced recruitment of DNA ligase III/XRCC1 to DNA strand breaks [214]. Later, an analogous influence of FUS on the maintenance of DNA integrity of mitochondrial DNA was demonstrated through FUS interaction and recruitment of mitochondrial DNA ligase IIIα to damage sites within mitochondrial DNA [215].

A recent study has provided a model for the hierarchical assembly of DSB condensates and the recruitment of effector proteins for DNA damage repair [212]. The authors show that functional sites of DSB repair are formed by co-condensation of PARP1 multimers with damaged DNA. The co-condensates exert mechanical forces to hold DNA ends together and become enzymatically active for PAR synthesis. PARylation promotes the release of PARP1 from DNA ends and the recruitment of effector proteins, such as FUS, which prevent the broken ends of DNA from separating.

#### 4.2.2. TDP-43

TDP-43 is a protein implicated in ALS. TDP-43, a 43 kDa protein, contains a nuclear localization signal, two RNA recognition motifs, and a prion-like domain [224]. TDP-43 is normally present in the nucleus, but in more than 95% of ALS patients, TDP-43 translocates from the nucleus, accompanied by its phosphorylation and subsequent formation of protein aggregates in the cytoplasm of neurons and glia [179,201,207,224]. In addition to ALS, TDP-43 is a hallmark of several NDs, including FTD, AD, and HD [220,224]. In mammalian neurons, TDP-43 has been shown to localize to transcriptionally active sites, including the nucleolus, suggesting a role in mRNA processing [179]. ALS neurons with loss of nuclear TDP-43 function have increased levels of DNA damage [221]. TDP-43 has been identified by proteomic analysis in mammalian cells as an interacting partner of Ku70 (subunit of the Ku antigen), the DSB sensor involved in NHEJ [276], but the role of TDP-43 in DNA repair remains unclear.

The first evidence for direct involvement of TDP-43 in the NHEJ-mediated DSB repair pathway was reported in [223]. Analysis of DSB repair kinetics in genomic DNA by neutral comet assay showed that repair was significantly delayed in TDP-43-depleted cells treated with the DSB-inducing agents, etoposide or bleomycin. Furthermore, the induction of DSBs led to an increased association between TDP-43 and the DDR markers γH2AX, pATM, and p53BP1 and the proteins of the NHEJ machinery, Ku antigen, DNA-PKcs, DNA polymerase λ, and DNA ligase 4/XRCC4 complex. Live cell imaging of TDP-43-depleted cells showed a slower disappearance of 53BP1 foci compared with controls, confirming slower DSB repair. A specific association of TDP-43 with the DNA ligase 4/XRCC4 complex, but not with DNA ligase 3/XRCC1, was found to be consistent with the involvement of TDP-43 in classical rather than alternative NHEJ. Interestingly, upon DSB induction in genomic DNA, a mutant TDP-43 that is mislocalized to the cytoplasm is able to trap the DNA ligase 4/XRCC4 complex, thereby preventing its translocation to the nucleus [219]. TDP-43 can recruit the ligase IV-XRCC4 complex to DSB sites and stimulate ligation in neuronal cells [109].

A comprehensive study [222] has demonstrated the multiple roles of TDP-43 in DSB repair. Overexpression of wild-type TDP-43 plays a protective role against DNA damage induced by etoposide or H2O2, whereas ALS-associated TDP-43 mutants lack this protective function. Using specific reporter assays, TDP-43 was shown to function in total NHEJ but not in the alternative pathway of NHEJ, supporting a role for TDP-43 in classical NHEJ. TDP-43 was also shown to be involved in the phosphorylation of γH2AX, further implicating TDP-43 in DNA damage signaling. It has also been shown that DNA damage induction leads to the mislocalization of TDP-43 to the cytoplasm, where it is localized in stress granules. In addition, the inhibition of classical NHEJ induces TDP-43 mislocalization to the cytoplasm.

The role of MMR in nondividing cells, particularly neurons, is not clear and may be limited to the repair of newly deaminated nucleobases, but it is of interest in the context of neurodegeneration as it may contribute to the expansion of trinucleotide repeats similar to those found in HD cells. TDP-43-mediated control of the expression of key MMR genes, MLH1, MSH2, MSH3, MSH6, and PMS2, has been demonstrated [139]. Depletion or overexpression of TDP-43 results in decreased or increased levels of RNA transcripts [139]. Furthermore, the protein–protein interaction between TDP-43 and the MMR factors MLH1 and MSH6 was shown to be dependent on DNA damage induced by methylmethanesulfonate [138].

#### 4.2.3. C9orf72

The G4C2 repeat expansion in the first intron of the C9orf72 gene (chromosome 9 open reading frame 72) is known to be the most common cause of ALS and FTD [170,225,230]. The C9orf72 repeat expansion is associated with other neurological disorders, including Alzheimer’s disease, multiple system atrophy, Huntington’s disease, cerebellar ataxia, multiple sclerosis, Parkinson’s disease, bipolar disorder, and schizophrenia [232]. The pathological mechanisms of these repeat expansions may be related to the formation of abnormal nucleic acid structures such as hairpins, G-quadruplexes, and R-loops [232]; disruption of the normal transcription of C9ORF72, leading to reduced C9ORF72 mRNA and protein in the frontal cortex and spinal cord of patients [170,225]; gain of toxicity through the unconventional translation of RNAs containing repeats, resulting in the generation of five different proteins with dipeptide repeats (DPRs), with polyPR (proline:arginine) being the most toxic [170,225,231]. However, the molecular mechanisms linking C9orf72 mutation and DNA repair remain incompletely understood [170,226,230,232]. Recent intensive studies reveal several underlying mechanisms [170,226,230]. Using appropriate DNA DSB repair assays, the authors estimated the efficiency of specific repair pathways and found that polyPR, polyGR (glycine:arginine), and polyGA (glycine:alanine) decreased the efficiency of NHEJ, single-strand annealing (SSA) and microhomology-mediated end joining (MMEJ) but not HR [226]. PolyPR was shown to inhibit DNA DSB repair by binding to the nucleolar protein nucleophosmin (NPM1), which is known to facilitate DNA repair [226].

Using confocal and super-resolution immunofluorescence microscopy, levels of RAD52, a component of the SSA repair machinery, were found to be increased in model neurons compared with isogenic cells in which the C9ORF72 expansion had been deleted by CRISPR/Cas9-mediated genome editing [226]. In addition, increased interaction between APE1 and NPM1 was demonstrated in C9orf72 patients compared with controls [229,232].

Overexpression of polyPR causes substantial DNA damage in cultured cells, primary cortical neurons, and the motor cortex of a polyPR transgenic mouse model [170]. A link was found between polyPR and FUS, another ALS-related gene product involved in DNA repair [170]. PolyPR is able to interact with FUS both in vitro and in vivo and is phase-separating with FUS [170]. In addition, polyPR disrupts the recruitment of FUS and its downstream protein XRCC1 to DNA damage sites induced by microirradiation [170]. Another study showed that polyGR synthesis in C9orf72 mutant cells is sufficient to promote aggregation of endogenous TDP-43; polyGR-mediated sequestration of full-length TDP-43 induces the formation of cytoplasmic TDP-43 inclusion bodies in an RNA-independent manner [228]. In the search for genetic modifiers of polyGR toxicity, Ku80 expression was shown to be significantly higher in Drosophila melanogaster flies expressing polyGR and in C9ORF72 iPSC-derived patient neurons [227], leading to increased levels of phosphorylated ATM and P53 and other downstream proapoptotic mediators. The increase in Ku80 levels was prevented by CRISPR-Cas9-mediated deletion of the expanded G4C2 repeats. Interestingly, partial inhibition of Ku80-dependent DNA repair by CRISPR/Cas9 or sRNA-mediated techniques suppressed apoptotic cell death.

A recent comprehensive study demonstrated the direct involvement of C9orf72 in different stages of NHEJ [230]. In response to DNA damage induced by laser microirradiation, C9orf72 localizes to the nucleus and is rapidly recruited to sites of DNA damage, colocalizing with the DNA DSB marker γH2AX. C9orf72 directly interacts with the catalytic subunit of DNA-dependent protein kinase and can disrupt DNA-PK complex assembly. In addition, C9orf72 promotes the recruitment of the ligase 4/XRCC4 complex to DNA damage sites. C9orf72 deficiency resulted in impaired NHEJ and accumulation of DNA damage. Furthermore, accumulated DNA damage in C9orf72-deficient neurons with polyGR expression led to neuronal loss through PARP1 overactivation. Thus, these results confirm a pathological mechanism in which C9orf72 deficiency synergizes with polyGR-induced DNA damage accumulation and PARP1 overactivation in ALS/FTD patients with mutated C9orf72.

#### 4.2.4. α-Synuclein

α-Synuclein, a 140-amino-acid-long, intrinsically disordered protein normally found in presynaptic nerve terminals and nuclei, is known to form aggregates in Parkinson’s disease, Parkinson’s dementia, and Lewy body dementia [209,242,243,244]. ND-associated aggregates of αSyn are phosphorylated at serine-129 (αpSyn), whereas normal αSyn protein is not. The nuclear functions and mechanisms by which αSyn exerts its neurotoxic effects are not fully understood. In vitro assays show that both αSyn and αpSyn can bind DNA by major groove interaction, with little requirement for DNA sequence [243]. Based on electrophoretic mobility shift assay and atomic force microscopy data, it was proposed that multiple αSyn molecules bound to DNA stabilize DNA in a bent conformation, whereas phosphorylation reduces the ability of αSyn to both bind and bend DNA. A landmark study [209] found that preformed αSyn fibrils (PFFs), derived from purified recombinant αSyn and similar to those found in PD patient cells, are capable of causing neuronal death via parthanatos, a cell death process dependent on PARP1 activation. Pathological αSyn was found to activate nitric oxide synthase, which leads to increased DNA damage through PARP1 activation and PAR synthesis; PAR generation accelerates the formation of pathological αSyn. αSyn PFF-induced neurotoxicity is reduced by PARP1 depletion or pharmacological inhibition.

Subsequently, using a variety of approaches (ICC, nuclear fractionation, Western blotting and neutral comet assay, and αSyn depletion silencing) in both human cells and a mouse model, direct evidence for the involvement of αSyn in the repair of induced DSBs was obtained [242]. Specifically, αSyn binds DS DNA and facilitates the T4 DNA ligase-mediated end-joining reaction. αpSyn is rapidly recruited to DNA damage sites in living mouse cortex. αSyn colocalizes with γH2AX (DSB marker) and PAR, the polymer involved in both single- and double-strand break repair. Depletion of αSyn in human cells leads to increased DSB levels after bleomycin treatment and a reduced ability to repair these lesions. Neurons containing αSyn inclusions show increased levels of DSBs in both mouse models and PD-patient-derived tissue. It has been proposed that aggregation of αSyn in the cytoplasm reduces its nuclear levels, leading to increased DSB levels [242]. Furthermore, using a specific plasmid-based NHEJ assay, a significant difference was found between WT and αSyn knockout cells, demonstrating that loss of ⍺Syn impairs NHEJ repair efficiency [244]. Taken together, these findings suggest that αSyn plays an important physiological role in DSB repair, although further studies are required to elucidate the exact molecular mechanism.

#### 4.2.5. Tau Protein

The two main neuropathological hallmarks of Alzheimer’s disease are intracellular neurofibrillary tangles (NFTs) composed of hyperphosphorylated tau and extracellular amyloid β peptide plaques [237,245,247,251,277].

Tau protein is mainly found in cells of the central nervous system, neurons, and oligodendrocytes. Tau binds and stabilizes microtubules in cells [250]. The tau sequence is enriched in polar and charged residues, which are distributed as follows: the N-terminus is negatively charged, while positively charged amino acids are concentrated in the middle and C-terminal regions. The lack of defined folding and charge distribution in tau gives it the ability to form weak multivalent interactions, predisposing it to LLPS, which can be enhanced by phosphorylation [246,249].

Tau is extensively regulated by phosphorylation; the longest isoform of the tau polypeptide contains more than 80 potential phosphorylation sites. LLPS of tau can occur in the presence of RNA, polyanions, and crowding agents in vitro at physiological protein concentrations [246,249]. Tau is able to interact with a wide range of cellular RNAs, including tRNAs, small nuclear RNAs, small nucleolar RNAs, mRNAs, and can bind DNA both in vitro and in cells, but the exact mechanism of tau function in nuclei remains to be elucidated [246,249].

Recently, the involvement of the tau protein in DSB repair has been demonstrated [248]. In response to etoposide-induced DSBs, cytosolic nonphosphorylated tau (non-p-tau) accumulates around nuclei together with tubulin, followed by conversion of tau to the phosphorylated form; knockdown of endogenous tau exacerbates the DSB situation in neurons, suggesting a protective role for tau.

Interestingly, under conditions of simultaneous DSB formation and microtubule disassembly, aberrant p-tau aggregation is dramatically enhanced. Taken together, these data suggest a central role for DSBs in tau-induced pathology in AD and that failure of DSB repair may be the cause of tauopathy [247,248]. In addition to direct involvement in DNA repair, the tau protein may exert its effect by interacting with TDP-43 in AD, ALS, and FTD [251,252,253].

#### 4.2.6. Amyloid β

Extracellular/intracellular β-amyloid peptide (Aβ) deposition is characteristic of Alzheimer’s disease. Aβ refers to peptides of about 40 amino acids (about 4 kDa) that result from cleavage of the Aβ precursor protein by β-secretase and γ-secretase, with the most abundant isoforms containing 40 and 42 amino acid residues, known as Aβ40 and Aβ42, respectively [184,185]. Aβ has a heterogeneous charge distribution, and its hydrophilic/hydrophobic balance can cause LLPS under certain conditions [184,185]. During the aggregation process, monomeric Aβ peptides first form soluble oligomeric intermediates, which then associate into high molecular weight assemblies (protofibrils), followed by the formation of amyloid fibrils and plaques. In the brain, both neurons and oligodendrocytes produce Aβ. Aβ is a multifunctional peptide involved in learning and memory, angiogenesis, neurogenesis, repair of blood–brain barrier leakage, and antimicrobial and antitumor effects [238,239]. Several functions of Aβ are associated with its extracellular and extra-nuclear location, while the nuclear function of Aβ remains enigmatic.

Data on the nuclear functions of β-amyloid peptides are rather limited. Reduced NHEJ activity and DNA-PKcs and Ku protein levels have been shown in AD brains [241]. Aβ is able to over-activate PARP1, which can lead to neuronal cell death [234]. Several papers have reported the involvement of Aβ in DNA repair of nuclear and mitochondrial DNA [233,236,237]. Aβ exerts its negative effect on NHEJ [236,237] and BER [235,237] via different molecular mechanisms. The aggregated Aβ fragment, Aβ(25–35), which has the same toxicity as the full length, inhibits DNA-PK in cells by down-regulating DNA-PKcs levels caused by oxidative stress [237]. Analysis of 8-oxo-dG levels in cells treated with Aβ(25–35) demonstrated the ability of the peptide to induce DNA base damage in differentiated neurons and to increase levels of APE1, which lacks its N-terminal domain [233].

It has also been shown that exogenous Aβ(1–42) oligomers are able to enter the cell nucleus, accumulate in insoluble oligomeric form, and inhibit DNA-PK activity [237]. Another study showed that treatment of cells with oligomers of Aβ(1–42) also triggers ROS production, which induces DNA damage in mitochondria and degrades DNA end-joining activity by NHEJ [236]. Taken together, these results demonstrate a negative impact of Aβ on NHEJ and BER, the predominant DNA repair pathways in post-mitotic neurons. It should be noted that this effect on DNA repair does not involve Aβ interaction with DNA but is realized at the protein level.

#### 4.2.7. NONO and SFPQ

Non-POU domain-containing octamer-binding protein (NONO) and splicing factor proline- and glutamine-rich (SFPQ) are members of the DBHS family of proteins, RNA-binding proteins with numerous vital functions, including involvement in DNA repair [264,266,267,268]. These proteins have attracted particular attention from researchers due to their emerging roles and implications in ND [268]. In addition to RNA recognition motifs (RRMs) responsible for DNA/RNA recognition/binding, both NONO and SFPQ contain low-complexity prion-like domains that enable the proteins to undergo LLPS [262,263,268]. The NONO protein was identified as a PAR-binding protein with the RNA recognition motif 1 (RRM1) responsible for PAR binding [267]. The in vivo recruitment of NONO to DNA damage sites is completely dependent on PAR. Upon PAR-dependent recruitment, NONO stimulates NHEJ and represses HR in vivo. An in vitro assay also confirmed the involvement of NONO in NHEJ [264]. NONO promotes the activity of DNA-PK through its ability to LLPS; it stimulates cell survival after exposure to ionizing radiation [262].

It was found that the SFPQ-NONO complex purified from HeLa cells and Ku antigen binds DNA independently, whereas the Ku protein requires free DNA ends for binding, while SFPQ-NONO does not. Both Ku and SFPQ-NONO have the ability to capture a second DNA fragment once bound to a DNA molecule. These findings, together with other data, suggest that NONO promotes end joining by binding to internal DNA sequences and, in cooperation with other repair proteins, by stabilizing a synaptic pre-ligation complex [269]. In vitro, a complex of SFPQ and NONO can replace the core factor of classical NHEJ, XLF, and promote this process [266]. At the same time, shRNA-mediated knockdown experiments indicate that both NONO and XLF are required in cells for efficient end joining. In addition, knockdown of NONO sensitizes cells to the interstrand cross-linking agent cisplatin [266]. A recent study has unexpectedly shown that the proteins NONO and SFPQ form inclusions in the nucleus of cells derived from PD and DLB patient material [263]. PD and DLB are known as synucleinopathies that contain inclusions of αSyn. Interestingly, these inclusions do not colocalize with Lewy bodies and accumulate at levels comparable to αSyn. Although the involvement of SFPQ in AD, FTLD, and ALS has been previously documented, the NDs are associated with pathological condensations of FUS and TDP-43 proteins [265,268].

#### 4.2.8. Huntingtin

Huntington’s disease (HD) is caused by a cytosine–adenine–guanine (CAG) expansion in exon 1 of the huntingtin (Htt) gene, which encodes mutant huntingtin (mHtt) characterized by an abnormal number of polyglutamine (polyQ) repeats at the N-terminus. How Htt causes neurotoxicity in HD remains unclear. The functions of normal Htt are not well understood [254]. Htt is highly expressed in neurons and is involved in axonal transport [254]. When the polyQ tract exceeds 35 repeats, mHtt misfolds to form intracellular aggregates. Interestingly, mHtt inclusions were found to co-localize with phosphorylated TDP-43 aggregates in the brains of HD patients [255]. In human cell cultures, expression of mHtt with more than 80 polyQ repeats leads to aggregation of endogenous TDP-43, while nonpathogenic Htt with 25 repeats has no effect [255].

Mutant Htt expression in neurons was shown to cause DSBs in genomic DNA and further promote DSB formation by impairing NHEJ through direct interaction with the Ku70 protein [256,261]. Ku70 supplementation rescued the phenotypes of a mouse HD model [256,261]. Htt forms a transcription-coupled DNA repair complex with RNA polymerase II subunit A, ataxin-3, PNKP, and cyclic AMP response element-binding protein. The functional integrity of this complex is compromised by mHtt, resulting in persistent accumulation of DNA breaks, preferentially in actively transcribed genes [257]. In addition, the direct involvement of Htt in response to oxidative DNA damage and its repair has been found [258,259].

Recently, it has been reported that Htt promotes the organization of the complex of PNKP, Ku70/80, and XRCC4 with the chromatin remodeler Brahma-related Gene 1 (BRG1) and stimulates the repair of transcription-associated DSBs in the brain [129]. Specifically, recruitment of Htt to DSBs in transcriptionally active regions was shown to be BRG1-dependent, whereas efficient recruitment of TA-EJ proteins is Htt-dependent. Mutant Htt impairs the interactions within this TA-EJ complex and DSB repair activity, resulting in the accumulation of DS breaks in HD tissues.

The mechanisms of HD development with analysis of genes and molecular pathways involved in pathogenesis are reviewed in detail in [260]. The authors note that DNA repair pathways involving PARP enzymes play an important role in HD pathogenesis and that the interactomes of mHTT-PARP1 can be considered therapeutic targets.

## 5. Conclusions

In this review, we summarized recent data on the involvement of proteins associated with neurodegenerative diseases in DNA repair in nervous system cells.

First, we gave a brief overview of the data on different DNA repair pathways (Figure 1), with a more detailed description of DNA base excision repair (Figure 2) and nonhomologous end joining (Figure 3), the repair pathways that function efficiently in post-mitotic neurons. DNA repair pathways in NS are largely similar to those in other tissues with some specific features. The emphasis on BER in neurons is related to the high level of oxidative lesions as the predominant form of DNA damage in NS, resulting from the high metabolic activity of the neuronal cells. Another interesting issue is the way (unique to neurons) in which DSBs are formed as a result of their normal physiological activity. Interestingly, due to the high transcriptional activity in neurons, subpathways of BER (TC-BER) and DSBR (TA-EJ) appear to be more active than in other somatic cells. As far as TA-EJ function is concerned, there is no established view on the molecular mechanism of this process and the protein composition of TA-EJ ensembles. Further studies are therefore needed to elucidate the requirements for this process.

We reviewed the data on the characteristics of PARP1 and ND-associated proteins, FUS, TDP-43, C9ORF72, Aβ, αSyn, Tau, Htt, and NONO/SFPQ (Table 2), which have the ability to undergo liquid–liquid phase separation, and experimentally confirmed their influence on DNA repair. Misfolding with the formation of aggregates (inclusions) has been reported for all of the above ND-associated proteins. This misfolding (aggregation) has been shown to abolish the positive influence of the proteins in DNA repair and, in some cases, even lead to the generation of DNA damage. The association of PARP1 with NDs is mainly related to its position at the crossroads of different DNA repair pathways. In addition, PARP1 is involved in the formation of PAR-dependent biomolecular condensates at sites of DNA damage. These condensates are enriched in DNA repair core proteins and facilitate DNA repair. PAR-dependent recruitment to DNA damage sites has been demonstrated for a number of ND-associated proteins, including FUS, TDP43, and NONO. Another interesting feature of PARP1 is that this enzyme generates most of the PAR in cells, the polymer that facilitates LLPS and appears to be involved in condensate formation at DNA repair sites. In addition to its positive role in organizing DNA repair, PAR is involved in neuronal death via parthanatos. Interestingly, PAR has been shown to facilitate the formation of pathological αSyn fibrils, which in turn can indirectly activate PARP1, leading to an increase in PAR levels [209]. Pathologic mislocalization and aggregation of the proteins in the cytoplasm and outside the cells reduce their nuclear levels, leading to less efficient DNA repair. This mechanism has been proposed for αSyn [242]. Mutant TDP-43 that is mislocalized to the cytoplasm is able to trap the DNA ligase 4/XRCC4 complex, thereby reducing its concentration in the nucleus [219]. TDP-43 can recruit the ligase IV-XRCC4 complex to DSB sites and stimulate ligation [109]. These examples demonstrate the diverse effects of these proteins in DNA repair, which requires a comprehensive study of their action. A better understanding of the basic mechanisms is of great importance for the prevention of neurodegeneration and the treatment of neurodegenerative diseases.

## Figures and Tables

**Table 1:** Human neurological diseases associated with mutated DNA repair genes in the rodent models.

Human Neurological Syndrome	Mutated Gene	Rodent Models	References
**Nucleotide excision repair**			
Cockayne syndrome: progressive neurodegeneration	*XPB*	Xpa and Xpb^XPCS^ double mutants: CS-like symptoms, including neurological defects	[15,143]
	*ERCC6/CSB*	Csb mutants mimicking human CS1AN allele: minor neurologic abnormalities	[15,144]
Xeroderma Pigmentosum (XP): part of XP patients develops neurological symptoms including microcephaly, mental retardation, cerebellar ataxia and peripheral neuropathy	*XPA-XPG*	Xpa^−/−^; Csb^−/−^ or Xpc^−/−^; Csb^−/−^ double mutants: CS- and XP-like symptoms including ataxia, motor dysfunction, reduced cerebellar neurogenesis, and neurodegeneration	[145,146]
**Base excision repair/Single-strand break repair**			
Spinocerebellar Ataxia with Axonal Neuropathy (SCAN1): cerebellar atrophy	*TDP1*	Tdp1^−/−^ mutant: progressive cerebellar atrophy	[15,147]
Biallelic mutations in human XRCC1: ocular motor apraxia, axonal neuropathy, and progressive cerebellar ataxia	*XRCC1*	Xrcc1^−/−^ mutant: embryonic lethality, but double Parp1^−/−^_Xrcc1^−/−^ mutant: reduced loss of cerebellar neurons and ataxia i	[148,149]
Ataxia with oculomotor apraxia 4 (AOA4), microcephaly with seizures (MCSZ)	*PNKP*	Pnkp: sensitivity of the myelin-producing oligodendrocytes to PNKP loss and DNA damage accumulation	[150]
Alzheimer disease	*POLB*	Polβ knockdown models in an AD mouse: an increase in synaptic problems as observed in AD patients	[151]
Ligase 3 (no human syndrome)	*LIG 3*	Lig3^Nes-cre^ conditional inactivation in mouse NS: mtDNA loss leading to ataxia	[18,152]
**Double strand break repair**			
ATR-Seckel Syndrome: microcephaly, dwarfism	*ATR*	Atr^S/S^ mutant mimicking ATR-Seckel Syndrome: microcephaly	[15,153]
LIG4 Syndrome: microcephaly	*LIG4*	Lig4 mutant: p53 dependent apoptosis of post-mitotic neurons	[154]
Ataxia-telangiectasia (A-T): progressive cerebellar ataxia that develops into severe motor dysfunction	*ATM*	Atm^L2262P/L2262P^ mutant rats: neuroinflammation and neurodegeneration (hind-limb paralysis)	[155]
		Atm^−/−^ mice: microglia activation and mild cerebellar degeneration	[156,157]
		ATM^−/−^ mice: aberrant astrocytic morphology and alterations of vasculature both in cerebellum and the visual system; reduced myelin basic protein immunoreactivity and signs of inflammation in ATM-deficient cerebella and optic nerve	[158]
		Atm^−/−^ mice: lose the ability to induce apoptosis in differentiating neuronal cells, but not in proliferating precursor neuroblasts, in response to DNA damage induced by ionizing radiation	[159,160]

**Table 2:** The ND-associated proteins that are prone to aggregation (with a confirmed LLPS capability) and have proven effects on DNA repair.

Name	Associated Neurological Disorder	Associated DNA Repair Pathway	Subcellular Localization/MLO	References
FUS	ALS, FTLD	NHEJ	Nucleus Paraspeckle Nucleolus Cajal body Cytoplasm Stress granule Transport granule	[178,179,193,212,214,215,216,217,218]
TDP-43	ALS, FTLD, AD, PD, HD	NHEJ MMR	Nucleus Paraspeckle Nucleolus Cajal body Cytoplasm Stress granule Transport granule	[109,138,139,176,179,201,207,219,220,221,222,223,224]
C9ORF72	ALS, FTLD HD, PD	BER (SSBR)	Nucleus Cytoplasm (co-aggregates with TDP-43)	[170,225,226,227,228,229,230,231,232]
Aβ	AD	NHEJ	Nucleus Cytoplasm Cell surface	[184,185,213,233,234,235,236,237,238,239,240,241]
αSyn	PD	NHEJ	Nucleus Colocalized with γH2AX or PAR foci Cytoplasm Presynaptic terminals	[183,206,209,242,243,244]
Tau	AD, ALS, FTLD	NHEJ	Nucleus Cytoplasm Axon, Dendrite, Cell membrane	[182,245,246,247,248,249,250,251,252,253]
Htt	HD	BER	Nucleus Cytoplasm	[129,254,255,256,257,258,259,260,261]
NONO/SFPQ	AD, PD, DLB, FTLD	NHEJ	Nucleus Paraspeckle	[262,263,264,265,266,267,268,269]

Amyloid β (Aβ); microtubule-associated protein (MAPT; Tau); α-synuclein (αSyn); TAR DNA-binding protein-43 (TDP-43); fused in sarcoma (FUS); non-POU domain-containing octamer-binding protein (NONO); splicing factor proline- and glutamine-rich (SFPQ); Huntingtin (Htt); Alzheimer disease (AD); amyotrophic lateral sclerosis (ALS); Huntington’s disease (HD); Parkinson’s disease (PD); PD and dementia with Lewy bodies (DLB); frontotemporal lobar degeneration (FTLD); single-strand break repair (SSBR, subpathway of BER).
